# Novel heterozygous compound *TRMT5* mutations associated with combined oxidative phosphorylation deficiency 26 in a Chinese family: a case report

**DOI:** 10.1186/s12887-022-03138-z

**Published:** 2022-02-02

**Authors:** Shuiyan Wu, Weixi Li, Zhenjiang Bai, Saihu Huang, Daoping Yang, Hongmei Chen, Ying Li, Ying Liu, Haitao Lv

**Affiliations:** 1 Pediatric Intensive Care Unit, Children’s Hospital of Soochow University, Suzhou, Jiangsu Province 215025 P.R. China; 2 Institute of Pediatric Research, Children’s Hospital of Soochow University, Suzhou, Jiangsu Province 215025 P.R. China; 3 Department of Cardiology, Children’s Hospital of Soochow University, Suzhou, Jiangsu Province 215025 P.R. China

**Keywords:** COXPD26, Medical exome sequencing, *TRMT5*, Mutation, Case report

## Abstract

**Background:**

Combined oxidative phosphorylation deficiency 26 (COXPD26) is an autosomal recessive disorder characterized by early onset, developmental delay, gastrointestinal dysfunction, shortness of breath, exercise intolerance, hypotonia and muscle weakness, neuropathy, and spastic diplegia. This disease is considered to be caused by compound heterozygous mutations in the *TRMT5* gene.

**Case presentation:**

In this study, we report a female child with COXPD26 manifesting as shortness of breath, gastrointestinal dysmotility, severe developmental delay, muscle hypotonia and weakness, exercise intolerance, renal and hepatic defects, and recurrent seizures with spastic diplegia. Interestingly, the hepatic feature was first observed in a COXPD26 patient. Medical exome sequencing with high coverage depth was employed to identify potential genetic variants in the patient. Novel compound heterozygous mutations of the *TRMT5* gene were detected, which were c.881A>C (p.E294A) from her mother and c.1218G>C (p.Q406H) and c.1481C>T (p.T494M) from her father.

**Conclusion:**

The newly emerged clinical features and mutations of this patient provide useful information for further exploration of genotype–phenotype correlations in COXPD26.

## Background

Combined oxidative phosphorylation deficiency 26 (COXPD26; MIM #616539), which includes developmental delay, shortness of breath, exercise intolerance, spasticity hyporeflexia, and hypotonia leading to the patient being unable to sit, stand, or walk without support, and decreased mitochondrial complex activity, is an autosomal recessive disorder characterized by a highly variable phenotype [1–3]. COXPD26 was first characterized in 1989 by Haller et al. [1]. To date, only four COXPD26 cases from three unrelated families are described [1–3]. Powell et al. reported a woman (the same case Haller et al. reported) and a 7-year-old boy, both of whom showed muscle weakness, exercise intolerance, neuropathy, gastrointestinal problems, spasticity with hyperreflexia, increased serum lactate levels, and decreased activity of mitochondrial complex IV. Over the following years, the woman developed exocrine insufficiency with malabsorption, liver cirrhosis, and renal tubulopathy. The boy showed additional features, including early-onset growth retardation, abnormal facial features, and hypertrophic nonobstructive cardiomyopathy. Mark reported two additional adult patients, both of whom had disease onset in childhood [2]. The patients presented similar aspects of the previously described phenotypes, including muscle weakness, exercise intolerance, neuropathy, and spastic diplegia [1–3]. Prematurity and the global developmental delay of one case were similar to the symptoms of the 7-year-old boy. Shortness of breath upon exertion was observed in Patient 1, as well as in the previously reported woman [2] (Table 1).

**Table 1:** Genetic and clinical features in individuals up to date

ID	Sex	TRMT5 Variations	Age at onset	Clinical course	Other features	References
1	female	c.312_315del; c.872G>A	childhood	Died at age of 55 years	life-long exercise intolerance, dyspnea, lactic acidosis, gastrointestinal issue with malabsorption, glucose intolerance, and pancreatic disorder, peripheral neuropathy, muscle weakness, renal tubulopathy, liver cirrhosis	[1]
2	male	c.312_315del; c.1156A>G	birth	Alive, 7 years old	premature delivery, growth retardation, gastrointestinal issue with intestinal pseudo-obstruction and poor feeding, cardiomyopathy, muscle hypotonia and weakness, demyelinating neuropathy, global development delay, lactic acidosis	[1]
3	female	c.312_315del c.872G>A	childhood	Alive, 46 years old	life-long exercise intolerance, muscle weakness, spasticity, axonal sensory neuropathy	[2]
4	female	c.312_315del c.872G>A	childhood	Alive, 51 years old	premature, global developmental delay, spasticity, progressive visual loss, frequent urinary tract infections, seizures	[2]
5	female	c.881A>C , c.1218G>C and c.1481C>T	birth	Died at age of 5 years	short of breath, gastrointestinal dysmotility, severe developmental delay, muscle hypotonia and weakness, exercise intolerance, renal and hepatic defect, recurrent seizures with spastic diplegia	This study

*TRMT5* (tRNA (guanine(37)-N1)-methyltransferase 5, NM_020810) is considered as the gene causing COXPD26 [1, 2]. *TRMT5* is located on chromosome 14q23.1 and encodes a 509-amino-acid protein that is produced in the cytosol and then transported into the mitochondria [2]. In mitochondria, it aids in the modification of tRNA 1-methylguanosine (m^1^G) at position 37, which is next to the 3′ end of the anticodon [4], both in vivo and in vitro [2]. Moreover, TRMT5 is not very specific with respect to the structure of bound tRNA which will be methylated [5]. In eukaryotes, about 11 kinds of tRNA can be modified with m^1^G37 [6, 7]. The methylation helps in reading frame maintenance by protecting against frameshifting when the peptides are elongated on the mRNA. Otherwise, a +1 frameshift error may occur, causing premature termination of protein synthesis [8]. It is conceivable that defective production of essential proteins due to errors such as frameshifts will disturb cellular function. For example, disruption of the *TRMT5* homologous gene TrmD in Salmonella typhimurium, Streptococcus pneumoniae, and Saccharomyces cerevisiae severely impairs microorganismal growth [9, 10]. In humans, when TRMT5 is transported into mitochondria, it methylates the tRNAs that are encoded by mitochondrial DNA (mtDNA) [11]. Altered tRNA methylation in mitochondria will affect the expression of proteins encoded by mtDNA and disturb respiratory function [1, 2]. This may be the pathogenic mechanism of COXPD26 caused by recessively inherited mutations in *TRMT5*. In this paper the first Chinese family with COXPD26 is described. We report a female patient with clinical features similar to previous cases of COXPD26 and a novel compound heterozygous mutation of *TRMT5*. The present study expands the currently available evidence concerning mutations associated with COXPD26.

## Case presentation

**Fig. 1:**
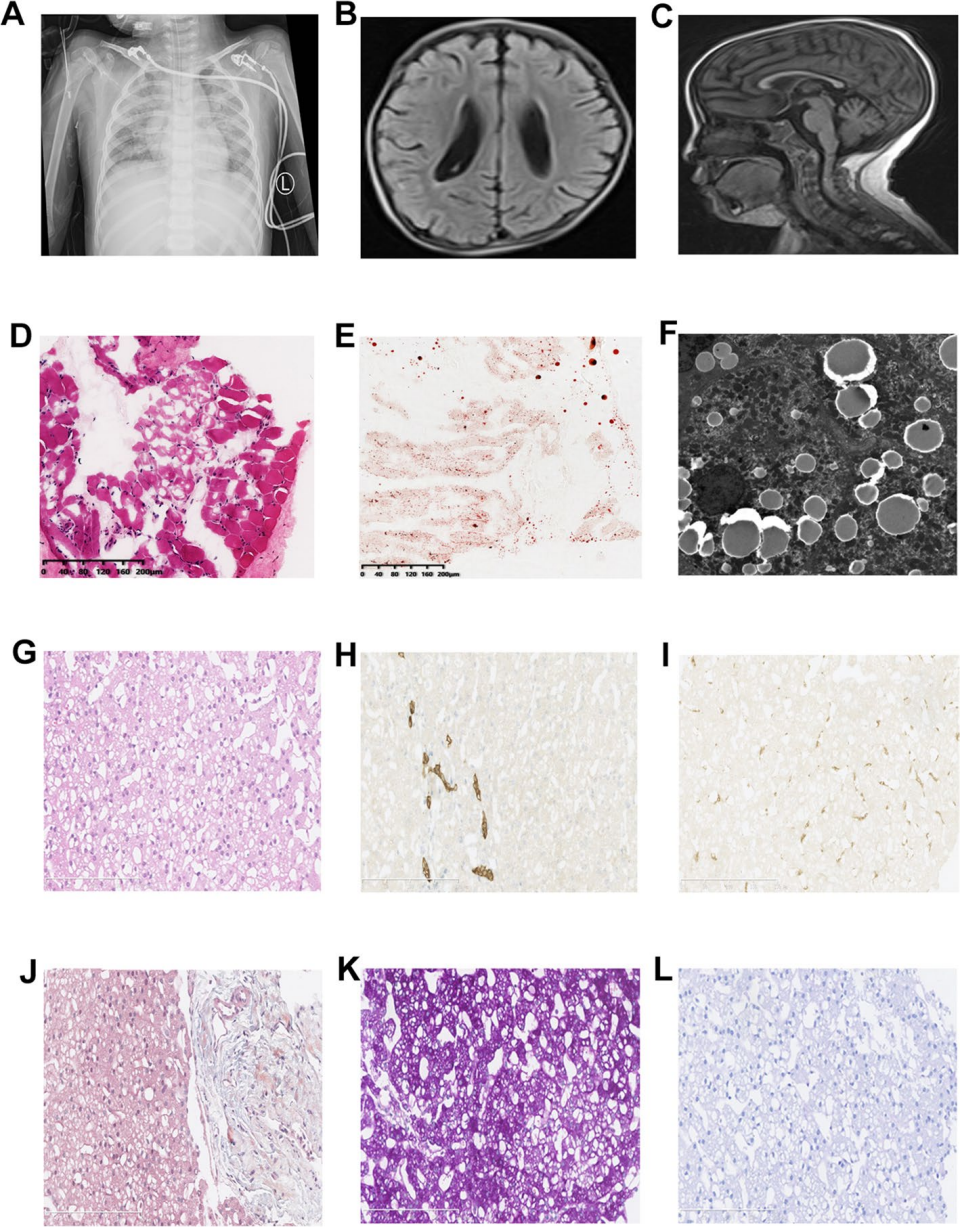
The clinical and histopathological examination. **A** Pneumonia indicated by X-ray result. **B** and **C** Abnormality in the brain with wider extracerebral space and thinner corpus callosum. **D** Myopathic changes with different size, small diameter of muscle fibers were observed by HE staining and **E** increased lipid droplets by oil staining (10×). **F** Hepatic biopsy showed abundant lipid droplets by electron microscopy. **G** Diffuse hepatocellular steatosis was observed in liver through light microscope; **H** Immunohistochemistry showed that the bile duct epithelium was positive for cytokeratin 19; **I** a few activated Kupffer cells were positive for CD68; **J** Masson staining showed fibrous hyperplasia in the portal area; **K** PAS reaction in liver was positive due to intracytoplasmic accumulation of glycogen; **L** D-PAS was negative in liver. All figures of liver by light microscope were showed with 20×

The pedigree underwent careful physical examination. The proband’s parents (I:1, I:2) were nonconsanguineous and both appeared normal. The proband (II:1) had frequently suffered from pneumonia since she was born, accompanied with shortness of breath much of the time (Fig. 1A). As a neonate, she presented laryngeal stridor and was diagnosed with laryngomalacia. She began to experience nausea and vomiting after being fed when she was 2 months old, and a gastrostomy tube was placed at that time. She presented considerable tympanites and gastrointestinal dysmotility with frequent vomiting. When she was 6 months old, she could raise her head. She could sit at the age of 10 months and stand at the age of 2 years. Developmental delay was obvious at the age of 4 years, when she reached a height of only 98cm (<2SD). Unfortunately, the patient suffered from motor retardation. Furthermore, she was unable to sit or stand without help, let alone walk. Moreover, muscular hypotonia and exercise intolerance was evident. Her motor development, speech, and social adaptation were assessed to be 2.5 years delayed. She only could communicate with her family by facial expressions, gestures, and simple sounds even though she had normal visual and auditory abilities. Recurrent seizures appeared with spastic diplegia at 5 years of age. A prominent fast wave in the frontal lobe was observed by electroencephalogram examination. Brain magnetic resonance imaging (MRI) showed a wide extracerebral space (Fig. 1B) and thin corpus callosum (Fig. 1C). Muscle biopsy showed myopathic changes with different size and small diameter of muscle fibers (Fig. 1D), and increased lipid droplets (Fig. 1E). Additionally, hepatic biopsy followed by electron microscopy revealed abundant lipid droplets (Fig. 1F). Through light microscopy, diffuse hepatocellular steatosis was observed (Fig. 1G). Immunohistochemistry showed that the bile duct epithelium was positive for cytokeratin 19 (Fig. 1H) and a few activated Kupffer cells were positive for CD68 (Fig. 1I), and Masson staining revealed fibrous hyperplasia in the portal area (Fig. 1J). Due to intracytoplasmic accumulation of glycogen, the periodic acid-Schiff (PAS) reaction was positive (Fig. 1K) and the D-PAS reaction was negative (Fig. 1L). In addition, blood tests indicated mildly elevated alanine aminotransferase levels and a physical exam did not indicate hepatomegaly. There was an apparent renal defect with oliguria and elevated creatinine, urea nitrogen, and uric acid levels, so dialysis was employed. Inherited metabolic disorders were screened, and slightly elevated lactate levels in blood and urine were observed. Serum antibody tests for toxoplasma, rubella virus, cytomegalovirus, and herpes simplex virus were negative and the patient’s plasma ammonia levels, triglyceride levels, blood glucose levels, karyotype, auditory brainstem responses, and thyroid function were all normal. There was no cardiac involvement apart from tachycardia; cardiac troponin I and creatine kinase isoenzyme levels were also in the normal range (Table 2). The patient died from stroke and respiratory failure at the age of 5 years. According to the patient’s main symptoms, which were in accordance with the common features of COXPD26, a diagnosis of COXPD26 was made.

**Table 2:** Laboratory results of the proband

Test	Results
Chromosome karyotype	46 XX, normal
Plasma ammonia	normal
Lactate	elevated (2.4–4.7mmol/L, normal range: less than 2.2 mmol/L)
TORCH	negative
Screening of genetic metabolism	slightly elevated lactate in blood and urine
Electroencephalogram	abnormal (prominent fast wave indicated in frontal region)
Auditory brain-stem responses	normal
Doppler ultrasound echocardiography	normal
Creatinine	elevated (202 μmol/L, normal range:45-84μmol/L)
Urea nitrogen	25.84 (2.9-8.2) mmol/L
Uric Acid	828.4 (155-357) μmol/L
Thyroid function	normal
Glutamic oxalacetic transaminase	396.7 (10-67) U/L
Glutamic pyruvic transaminase	100.2 (5-35) U/L
Bilirubin	normal
Cardiac troponin I	0.08(0.00-0.09) pg/ml
Creatine kinase isoenzyme	2.5(0.0-3.7) ng/ml
Myoglobin	77.4(11.6-73.0) ng/ml
Triglyceride	normal
Blood glucose	normal (4.7-7.5mmo/L)

*TORCH* serum antibody tests for toxoplasma, rubella virus, cytomegalovirus, and herpes simplex virus

**Fig. 2:**
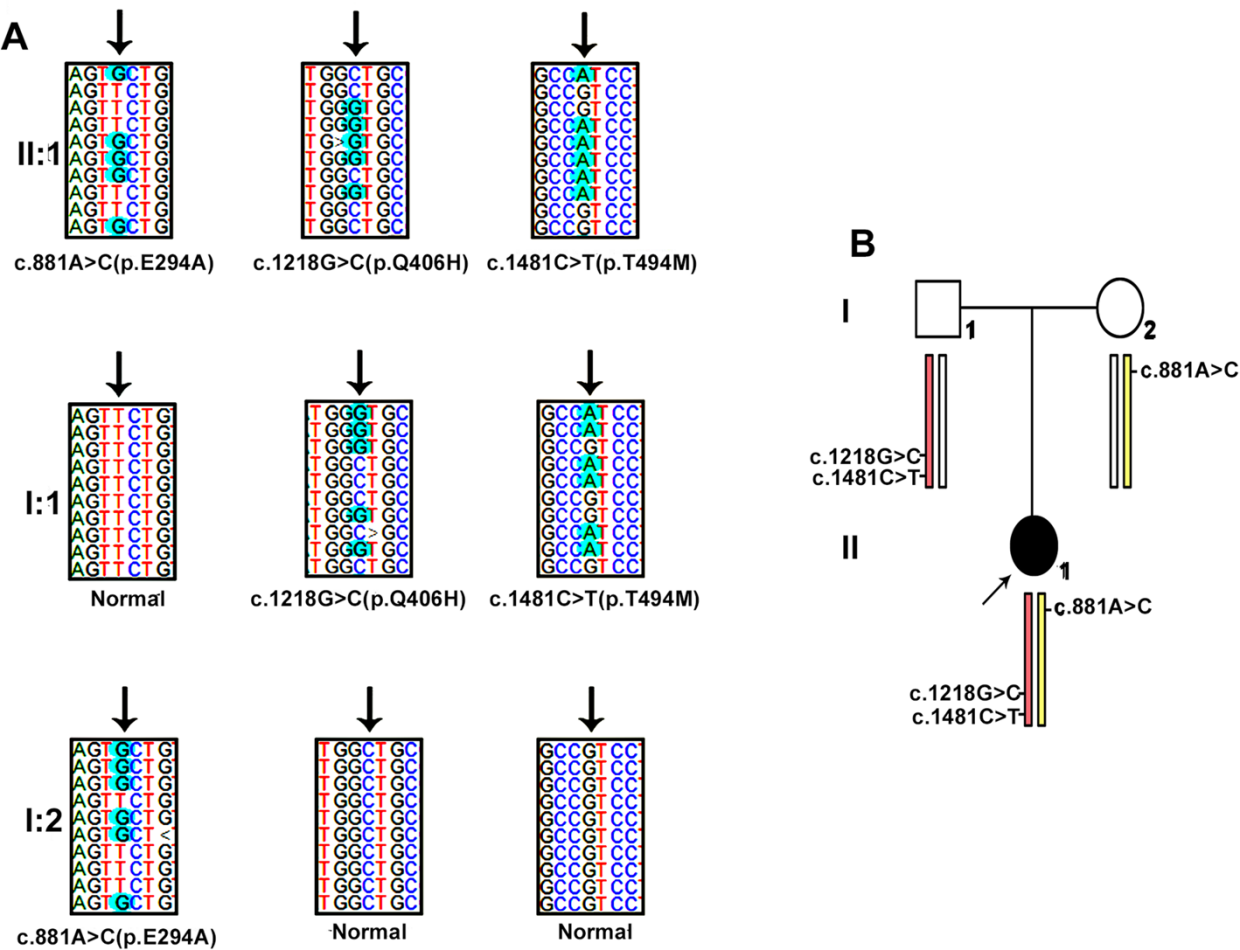
**A** The mutations in the pedigree. Three mutations identified in the proband (II:1). Two mutations identified in the father (I:1). One mutation identified in the mother (I:2). **B** The pedigree information. The proband (II:1) was indicated by the arrow. The parents (I:1, I:2) appeared normal

**Fig. 3:**
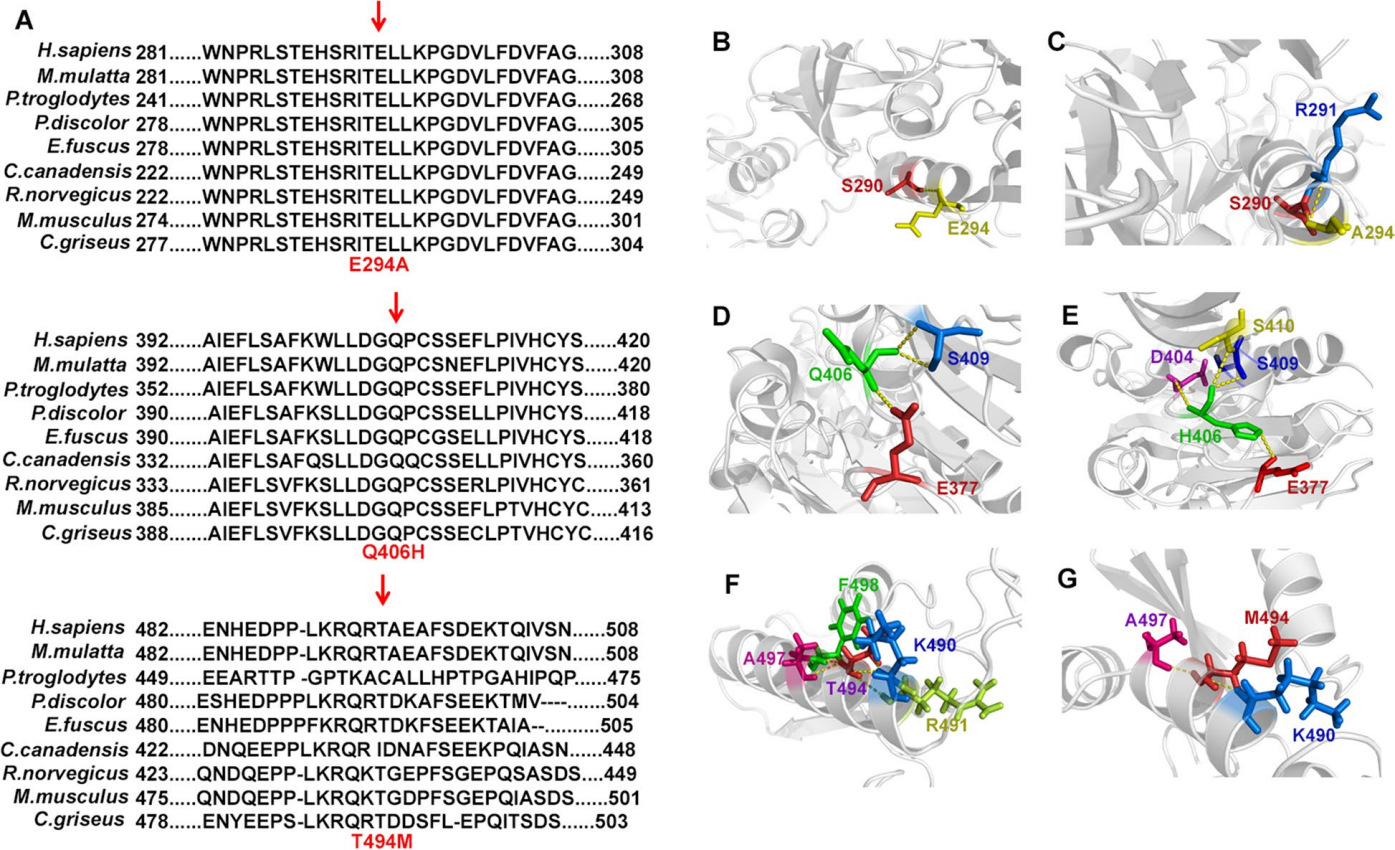
Genetic analysis of the mutations. **A** The multiple alignments of the TRMT5 amino acid sequences were performed with Clustalx (Ver. 1.83). The amino acid sequences were all from the NCBI public database (http://www.ncbi.nlm.nih.gov/protein). **B**, **C** There was one more hydrogen bond on Residue 294 when wild-type E was replaced by A. **D**, **E** There were two more hydrogen bonds on Residue 406 when wild-type Q was replaced by H. **F**, **G** There were two less hydrogen bonds on Residue 494 when wild-type T was replaced by M. The homologous models of truncated wild-type and mutated TRMT5 fragments including Residue 294 and 406 were generated in SWISS-MODEL (https://swissmodel.expasy.org/). And the homologous models of truncated wild-type and mutated TRMT5 fragments including Residue 494 was generated in QUARK (https://zhanglab.ccmb.med.umich.edu/QUARK2/),for SWISS-MODEL could only mimic the 3D structure of TRMT5 from Residue 96 to 469

This study was conducted in accordance with the Declaration of Helsinki and was approved by the ethics committee (Children’s Hospital of Soochow University, No. 2019LW010). Written consent was obtained from the parents before they participated in the genetic investigation. To achieve an accurate genetic diagnosis, medical exome sequencing was carried out with a trio sample strategy. The advantages and experimental procedures of this technique were previously described by our team [12]. Novel heterozygous missense mutations in *TRMT5* (NM_020810) were detected in the proband (Fig. 2), which were c.881A>C (p.E294A) from her mother and c.1218G>C (p.Q406H) and c.1481C>T (p.T494M) on the same chromosome from her father. The proband inherited these missense mutations in *TRMT5*, which made herself compound heterozygous. The c.881A>C (p.E294A) and c.1481C>T (p.T494M) mutations were reported with a minor-allele frequency of 0.0003 and 0.0008, respectively, in the Aggregation Consortium (ExAC) Browser. The c.1218G>C (p.Q406H) mutation had not been recorded in the ExAC Browser previously. The residue T494 was considered as a phosphorylated site according to the PhosphoSitePlus browser (https://www.phosphosite.org/homeAction.action). The three mutated residues were all evolutionarily well conserved (Fig. 3A), which indicates these residues have important functions.

To explore whether the mutations could lead to protein misfolding, homologous models of truncated wild-type and mutated TRMT5 fragments including each mutated residue were generated. The peptides containing p.E294A and p.Q406H were modeled on SWISS-MODEL (https://swissmodel.expasy.org/). As SWISS-MODEL could only build the model from residue 96 to 469, we built the peptide model from residue 406 to 509 (which contained p.T494M) on QUARK (https://zhanglab.ccmb.med.umich.edu/QUARK2/). The hydrogen bonds were analyzed by PyMOL (ver 1.4.1). There was one more hydrogen bond between R291 and the mutated residue A294 than between R291 and the wild-type E294 (Fig. 3B and C). Moreover, there were two more hydrogen bonds between the mutated residues H406 and D404 and S410 than between the wild-type Q406 and D404 and S410 (Fig. 3D and E), and there were two hydrogen bonds less between the mutated residues M494 and R491 and F498 than between the wild-type T494 and R491 and F498 (Fig. 3F and G). All of the alterations in hydrogen bonds might lead to misfolding of TRMT5 to affect its normal function. The bioinformatic analysis indicated these three mutations in TRMT5 were all associated with COXPD26.

## Discussion and conclusion

Our case presented recurrent pneumonia, shortness of breath, gastrointestinal dysmotility with frequent vomiting, severe developmental delay, muscular hypotonia and exercise intolerance, and renal and hepatic defects. She was unable to sit or stand without help. In addition, her motor development, speech, and social adaptation were delayed, which is consistent with previous reports [1–3]. Recurrent seizures with spastic diplegia appeared when she was 5 years old, which is consistent with Patient 2 in Mark’s article [3]. A wide extracerebral space and thin corpus callosum were indicated by brain MRI. Brain MRI of this disease is not specific, as each patient has different manifestations. Muscle biopsy showed myopathic features without ragged-red fibers, as was the case for the 7-year-old patient in Powell’s paper [2]. Muscle biopsy also indicated reduced respiratory chain complex enzyme activities. Unfortunately, the enzyme activity of COX could not be detected in our lab. However, liver pathology of this disease has not been reported in previous research. In the present study, light and electron microscopy indicated a large amount of glycogen and high fat accumulation in the liver, which likely led to liver damage with increased collagen deposition and Kupffer cell activation. Blood glucose levels were within the normal range, but the patient’s delayed motor development and exercise intolerance indirectly suggested mitochondrial complex deficiency, as previously reported in other cases [1–3], eventually leading to reduced glycogen utilization, followed by the conversion of excessive glycogen into fatty acid, which manifested as increased fatty acid levels in the liver and muscle tissue.

To some extent, our observations indicate novel features of this disease. Most of the clinical features, including early onset, muscle weakness, exercise intolerance, neuropathy, and spastic diplegia, were shared with previously reported patients. The manifestation of developmental delay, lactic acidosis, seizure, and gastrointestinal, renal, and liver problems also overlapped with previous cases [1–3].

There were four COXPD26 patients in previous reports, who all carried two compound heterozygous mutations/deletions in *TRMT5*. They shared one common deletion, c.312_315del, which leads to a frameshift [2, 3]. In our study, the proband had different compound heterozygous missense mutations of *TRMT5*: c.881A>C (p.E294A) from her mother and c.1218G>C (p.Q406H) and c.1481C>T (p.T494M) from her father. The parents appeared normal, whereas the proband had a severe syndrome and died from stroke and respiratory failure at the age of 5 years. To the best of our knowledge, these are the first *TRMT5* mutations identified in Asian COXPD26 patients. All previous COXPD26 patients had early symptom onset, but the patients lived longer than our proband and had a higher quality of life. Our case presented with severe symptoms at an early age and with different manifestations than the other COXPD26 patients, which may be the result of the genotype identified in this patient. Further analysis is necessary to confirm this hypothesis.

In the present study, medical exome sequencing was used to explore the possible genetic defects resulting in the disease. Compared to whole genome and whole exome sequencing, medical exome sequencing focuses on clinical interpretable regions of genes, which greatly improves the accuracy of sequencing and broadens the spectrum [12]. Given that the phenotype of the proband was much more severe than those in previous reports, we considered whether there was molecular “double trouble” in the patient’s genome. However, we did not find any known copy number variation which was associated with the phenotypes. We excluded any other candidate genes based on our whole exome sequencing results for either of the two reasons.

In conclusion, we identified novel heterozygous mutations of *TRMT5* that cause COXPD26 for the first time in a Chinese family. This study will further our understanding of the molecular mechanisms of COXPD26 and contribute to elucidation of the phenotype–genotype correlations of related disorders.

## Data Availability

The datasets used and/or analysed during the current study are available from the corresponding author (Haitao Lv and Ying Liu) on reasonable request.

## Abbreviations

COXPD26: Combined oxidative phosphorylation deficiency 26
TRMT5: tRNA (guanine(37)-N1 methyltransferase 5
MRI: Magnetic resonance imaging
ABR: Auditory brain-stem responses
MAF: Minor-allele frequency
CNV: Copy number variation
